# Co-occurrence of mutations in *NF1* and other susceptibility genes in pheochromocytoma and paraganglioma

**DOI:** 10.3389/fendo.2022.1070074

**Published:** 2023-01-25

**Authors:** Sara Mellid, Eduardo Gil, Rocío Letón, Eduardo Caleiras, Emiliano Honrado, Susan Richter, Nuria Palacios, Marcos Lahera, Juan C. Galofré, Adriá López-Fernández, Maria Calatayud, Aura D. Herrera-Martínez, María A. Galvez, Xavier Matias-Guiu, Milagros Balbín, Esther Korpershoek, Eugénie S. Lim, Francesca Maletta, Sofia Lider, Stephanie M. J. Fliedner, Nicole Bechmann, Graeme Eisenhofer, Letizia Canu, Elena Rapizzi, Irina Bancos, Mercedes Robledo, Alberto Cascón

**Affiliations:** ^1^ Hereditary Endocrine Cancer Group, Spanish National Cancer Research Centre (CNIO), Madrid, Spain; ^2^ Histopathology Core Unit, Spanish National Cancer Research Centre (CNIO), Madrid, Spain; ^3^ Anatomical Pathology Service, Hospital of León, León, Spain; ^4^ Institute for Clinical Chemistry and Laboratory Medicine, University Hospital Carl Gustav Carus, Medical Faculty Carl Gustav Carus, Technische Universität Dresden, Dresden, Germany; ^5^ Endocrinology Department, University Hospital Puerta de Hierro, Madrid, Spain; ^6^ Endocrinology and Nutrition Department, La Princesa University Hospital, Madrid, Spain; ^7^ Department of Endocrinology, Clínica Universidad de Navarra, Pamplona, Spain; ^8^ Hereditary Cancer Genetics Group, Vall d’Hebron Institute of Oncology (VHIO), Barcelona, Spain; ^9^ Department of Endocrinology and Nutrition, Hospital Universitario 12 de Octubre, Madrid, Spain; ^10^ Endocrinology and Nutrition Service, Reina Sofia University Hospital, Cordoba, Spain; ^11^ Department of Pathology, Bellvitge University Hospital, Centro de Investigación Biomédica en Red de Cáncer (CIBERONC), Barcelona, Spain; ^12^ Molecular Oncology Laboratory, Instituto Universitario de Oncologia del Principado de Asturias, Hospital Universitario Central de Asturias, Oviedo, Spain; ^13^ Department of Clinical Genetics, Erasmus Medical Center, Rotterdam, Netherlands; ^14^ Department of Endocrinology, William Harvey Research Institute, Queen Mary University of London, London, United Kingdom; ^15^ Pathology Unit , Department of Laboratory Medicine, Azienda Ospedaliero-Universitaria (AOU) Città della Salute e della Scienza di Torino, Torino, Italy; ^16^ Endocrinology Department, National Institute of Endocrinology, Bucharest, Romania; ^17^ First Department of Medicine, University Medical Center Schleswig-Holstein, Lübeck, Germany; ^18^ Department of Medicine III, University Hospital Carl Gustav Carus, Technische Universität Dresden, Dresden, Germany; ^19^ Department of Experimental and Clinical Medicine, University of Florence, Florence, Italy; ^20^ Division of Endocrinology, Metabolism and Nutrition, Mayo Clinic, Rochester, MN, United States; ^21^ Centro de Investigación Biomédica en Red de Enfermedades Raras (CIBERER), Instituto de Salud Carlos III, Madrid, Spain

**Keywords:** pheochromocytoma, NF1, germline mutation, DLST, MDH2, co-occurrent mutations

## Abstract

**Introduction:**

The percentage of patients diagnosed with pheochromocytoma and paraganglioma (altogether PPGL) carrying known germline mutations in one of the over fifteen susceptibility genes identified to date has dramatically increased during the last two decades, accounting for up to 35-40% of PPGL patients. Moreover, the application of NGS to the diagnosis of PPGL detects unexpected co-occurrences of pathogenic allelic variants in different susceptibility genes.

**Methods:**

Herein we uncover several cases with dual mutations in NF1 and other PPGL genes by targeted sequencing. We studied the molecular characteristics of the tumours with co-occurrent mutations, using omic tools to gain insight into the role of these events in tumour development.

**Results:**

Amongst 23 patients carrying germline NF1 mutations, targeted sequencing revealed additional pathogenic germline variants in DLST (n=1) and MDH2 (n=2), and two somatic mutations in H3-3A and PRKAR1A. Three additional patients, with somatic mutations in NF1 were found carrying germline pathogenic mutations in SDHB or DLST, and a somatic truncating mutation in ATRX. Two of the cases with dual germline mutations showed multiple pheochromocytomas or extra-adrenal paragangliomas - an extremely rare clinical finding in NF1 patients. Transcriptional and methylation profiling and metabolite assessment showed an “intermediate signature” to suggest that both variants had a pathological role in tumour development.

**Discussion:**

In conclusion, mutations affecting genes involved in different pathways (pseudohypoxic and receptor tyrosine kinase signalling) co-occurring in the same patient could provide a selective advantage for the development of PPGL, and explain the variable expressivity and incomplete penetrance observed in some patients.

## Introduction

Over the last two decades, the number of genes identified as involved in the hereditary susceptibility to developing pheochromocytoma (PCC) or paraganglioma (PGL), collectively known as PPGLs, has grown to more than fifteen, increasing the percentage of patients carrying known germline predisposing mutations to 35-40% (1). Furthermore, the prevalence and penetrance of PPGL-associated mutations is highly variable and parent-of-origin effects have been identified for at least, three susceptibility genes (*SDHD*, *SDHAF2* and *MAX*), making genetic diagnosis of the disease challenging. Comprehensive gene panels or exome sequencing, which allow the examination of all predisposing genes in one test, have replaced complicated algorithms employed for genetic testing of PPGL patients, and as a result of the shift from sequential gene testing to next-generation sequencing, unexpected genetic variants have been discovered. In this regard, two recent studies describe the presence of pathogenic germline variants in both *NF1* and *SDHD* genes in patients with multiple PPGLs (2, 3), one of whom had no clinical sign of neurofibromatosis type 1 (NF1; MIM #162200). The presence of germline variants in more than one susceptibility gene co-occurring in the same patient adds a further layer of complexity to the genetic diagnosis of the disease, and suggests that a more intricate genetic condition accounts for the development of PPGL in some NF1 patients.

Development of PPGL in patients with NF1 is thought to be very rare (0.1-5.7%) (4–6). A recent study revealed that only 0.3% (1/342) of patients with NF1 developed PPGL (7), while an exhaustive study for the presence of PPGL amongst patients diagnosed with neurofibromatosis (n=156), found that up to 7.7% of patients developed PPGL (8). This percentage could be underestimated since the use of targeted NGS has led to the identification of *NF1* germline mutations in PPGL patients without a clear clinical diagnosis of NF1 (3, 9–12). Nevertheless, the percentage of patients developing PPGL amongst NF1 patients contrasts with that observed for other PPGL syndromes, e.g. 20% in von Hippel-Lindau (VHL; MIM #193300) and 50% in multiple endocrine neoplasia type 2A (MEN2; MIM #171400) (13, 14). Moreover, PPGLs are multifocal or bilateral in 43-45% of VHL cases and in 50-80% of MEN2 cases, compared to 16% in NF1 patients. These differences point to still unknown mechanisms that could account for the development of PPGL in NF1 patients.

We recently identified three patients carrying *NF1* germline mutations and additional variants in other PPGL susceptibility genes. Interestingly, two of the three cases showed quite uncommon clinical findings (bilateral PCC and extra-adrenal PGL). Therefore, herein we analyse, using a comprehensive gene panel, a series of NF1 patients with PPGL, enriched in cases with multiple PPGLs, to investigate the co-occurrence of *NF1* mutations with causal variants in other PPGL susceptibility genes. Moreover, we study the molecular characteristics of the tumours with dual mutations, using various omic tools, to gain insight into the role of this genetic event in the development of PPGL.

## Methods

### Patients and samples

Twenty-three patients diagnosed with PPGL and carrying a germline *NF1* mutation were included in the study (**Table 1**). The series was enriched with patients developing bilateral PCC or extra-adrenal PGLs. In addition, three patients carrying known germline or somatic mutations in other PPGL susceptibility genes, and a somatic *NF1* alteration were also included for discussion. A summary of the clinical data of the patients included in the study is provided in **Table 2**. Genomic DNA was extracted from available peripheral-blood leukocytes with the Maxwell Blood DNA-purification system (Promega). Tumour DNA was obtained from all patients with the DNeasy Blood and Tissue kit (QIAGEN) for frozen tissue and the Covaris S2 System (Covaris) for formalin-fixed paraffin-embedded (FFPE) tissue according to the manufacturers’ instructions. Informed written consent was obtained for all participants in the study. Ethical approval was granted by local ethical committees with the following reference numbers: 13-004137, 06/Q0104/133, 15/024, 88/11, 2011/0020149, 14/11.06.2020, PI54_2016-v2.

**Table 1:** Molecular findings identified in the patients included in the study.

ID	*NF1* mutation	Varsome/ ClinVar	gnomAD frequency	*NF1* LOH/2^nd^ hit	2^nd^ mutation	Varsome/ ClinVar	gnomAD frequency	LOH
1^#^	p.(His2423GlnfsTer4)	NA; path	–	Yes	p.(Gly374Glu) DLST	18/20 path; path	2/251464	No
2	p.(Leu2323Pro)	19/20 path; VUS	–	Yes	p.(Lys314Met) MDH2	15/20 path; NA	6/251092	No
3	p.(Ser1561Ile)	15/20 path; NA	–	No	p.(Lys314Met) MDH2	15/20 path; NA	6/251092	No
4	p.(Ser1838TyrfsTer23)	NA; NA	–	–	–	–	–	–
5^#^	p.(Thr586ValfsTer18)	NA; path	1/251120	Yes	–	–	–	–
6^#^	p.(Ser260IlefsTer7)	NA; NA	–	Yes	–	–	–	–
7	c.5944-2A>G	6/6 path; path	–	Yes	–	–	–	–
8	c.586+1G>A	6/6 path; path	–	Yes	–	–	–	–
9	p.(Arg2258Ter)	7/8 path; path	–	No	–	–	–	–
10	p.(Met1?)	13/17 path; path	–	Yes	–	–	–	–
11	p.(Leu145GlnfsTer10)	–	–	Yes	–	–	–	–
12	p.(Tyr2285Ter)	7/8 path; path	2/282598	dubious	–	–	–	–
13	p.(Ile429AspfsTer2)	NA; NA	–	Yes	–	–	–	–
14	c.2851-6_2851-3del	NA; path	–	Yes	–	–	–	–
15	p.(His1826Arg)	19/20 path; VUS	–	No	–	–	–	–
16	p.(Gln2589Ter)	7/8 path; path	–	Yes	–	–	–	–
17	c.6147+1G>A	6/6 path; path	–	–	–	–	–	–
18	p.(Tyr628LeufsTer6)	NA; path	–	Yes	–	–	–	–
19	p.(Gln2373Ter)	7/8 path; path	–	Yes	–	–	–	–
20	p.(Arg681Ter)	7/8 path; path	1/250690	Yes	–	–	–	–
21	p.(Gln1086Ter)	7/8 path; path	–	Yes	–	–	–	–
22	c.6820-1G>T	6/6 path; NA	–	Yes	**p.(Arg16Ter) PRKAR1A**	7/8 path; path	–	No
23	p.(Gly2379Arg)	17/20 path; VUS	4/251372	No	**p.(Gly35Trp) H3-3A**	15/19 path; NA	–	No
24	**p.(Leu134GlnfsTer20)**	NA; NA	–	Yes	p.(Gly374Glu) DLST	18/20 path; path	2/251464	No
25	**c.4333-1G>A**	6/6; likely path	1/ 227062	Yes	c.423+1G>A *SDHB*	6/6 path; path	3/ 251374	No
26	**p.(Ser2181ValfrTer19)**	NA; path	–	–	**p.(Arg808Ter) ATRX**	5/5 path; NA	–	No

^#^, previously reported case; Path, pathogenic; VUS, variant of uncertain significance; NA, not available; LOH, loss of heterozygosity; bold letters, somatic mutation; we used the ENST00000358273 transcript to name the NF1 variants.

**Table 2:** Clinical data of the patients included in the study.

ID	Tumor	Distant metastasis	Age	Sex	FH PCC	Clinical features of NF1	FH NF1	Metanephrines
1^#^	Bilateral PCC & MTC	No	56	M	No	Café-au-lait spots, axillary and inguinal freckling, Lisch nodules, macrocephaly, intra- and extra arachnoidal neurofibromas	No	NM
2	PCC	No	25	M	Yes	–	Yes	M>NM
3	TA PGL	No	76	M	No	–	No	NA
4	PCC	No	39	M	No	–	No	NM>M
5^#^	Bilateral PCC	No	45	F	NA	Café-au-lait spots, skin fibromas, multiple GIST and axillary and inguinal freckling	NA	M&NM
6^#^	PCC	No	53	F	No	Unknown NF1 features	Yes	M
7	PCC	Yes	15	F	Yes	Skin neurofibromas	Yes	M
8	PCC	No	59	M	No	–	No	M>NM
9	PCC	No	56	M	No	–	No	M>NM
10	PCC	No	60	M	No	–	No	NM>M
11	PCC	No	43	F	NA	–	NA	NA
12	PCC	No	57	M	No	Neurofibromas, bilateral Lisch nodules, choroidal lesions	Yes	M>NM
13	Bilateral PCC	Yes	60	M	NA	Multiple neurofibromatoma over skin, freckles, intellectual disability (learning difficulties), hearing impairment	NA	M&NM
14	Bilateral PCC	No	56	F	NA	Café-au-lait spots, typical fibromas	NA	M
15	PCC	No	44	M	No	–	No	M
16	PCC	No	51	M	No	–	No	M
17	PCC	No	39	F	No	–	No	NM
18	Bilateral PCC	No	42	F	No	Café-au-lait spots, malignant peripheral nerve sheath tumour, neurofibromas	No	M
19	Bilateral PCC	No	42	F	No	Café-au-ait spots, axillary and inguinal freckling, neurofibroma	No	M&NM
20	Bilateral PCC	No	48	F	No	Café-au-lait spots, neurofibromas, inguinal freckling	No	M&NM
21	Bilateral PCC	No	30	F	No	Café-au-lait, deformity of left forearm, hyperpigmentation , delayed development, grand mal seizures, hamartoma, neurofibroma (plexiform), macrocephaly	No	D&M&NM
22	Bilateral PCC	No	41	F	Yes^‡^	Café-au-lait spots, neurofibroma, axillary freckling	Yes	M&NM
23	PCC	Yes	50	F	No	–	No	NM
24	PCC	No	55	F	No	–	No	M>NM
25	PCC	No	66	F	Yes	–	Yes	M
26	PCC	Yes	63	M	No	–	No	NM

^#^, previously reported case (ID 1: Gieldon et al. 2018; ID 5: Barbero et al. 2021; ID 6: Matas-Nadal et al. 2022); PCC, pheochromocytoma; TA, thoracic-abdominal; FH, family history; M, metanephrine; NM, normetanephrine; Path, pathogenic; VUS, variant of uncertain significance; NA, not available; LOH, loss of heterozygosity; ^‡^, suspicious but not confirmed; MTC, medullary thyroid carcinoma; GIST, gastrointestinal stromal tumor; PGL, paraganglioma; D, dopamine.

### Targeted next-generation sequencing (NGS) and loss-of-heterozygosity (LOH) analysis

**Figure 1:**
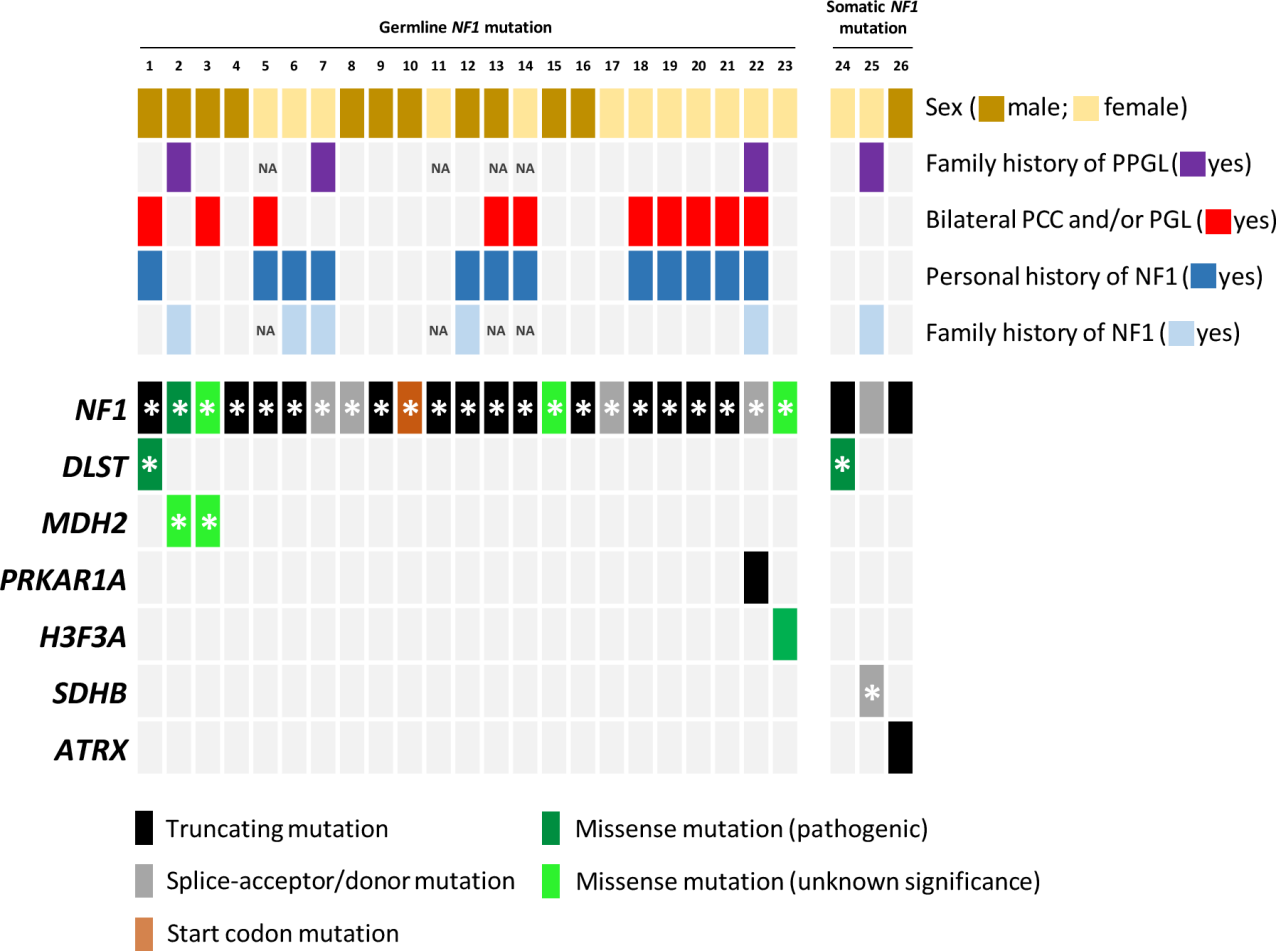
Mutational profile of the twenty-six samples included in the study. Each column represents a tumour, with coloured rectangles representing mutations that are germline (denoted with an asterisk) or somatic in each row. Missense variants that ClinVar does not classify as pathogenic mutations and/or that are absent in NF1 patients were categorized as variants of unknown significance. NA, not available.

DNA obtained from blood or tumour samples was used for targeted sequencing. The diagnostic PPGL target panel included all susceptibility genes described so far, and some genes mutated somatically in PPGL, in total 33 genes (*CSDE1*, *KIF1B*, *SDHA*, *SDHB*, *SDHC*, *SDHD*, *SDHAF1*, *SDHAF2*, *EGLN1*, *EGLN2*, *FH*, *MDH2*, *DLST*, *IDH2*, *IDH1*, *TMEM127*, *VHL*, *MET*, *RET*, *PTEN*, *HRAS*, *MEN1*, *KRAS*, *MAX*, *GOT2*, *NF1*, *PRKAR1A*, *ATRX*, *BRAF*, *EPAS1*, *SLC25A11*, *DNMT3A*, *H3F3A*). TruSight (Illumina) oligo probes were designed for the genes of interest. DesignStudio software (Illumina) was used for the design of 891 amplicons. Once the library was prepared, sequencing was performed using a MiSeq sequencer (Illumina). Alignment of sequences was done by MiSeq Reporter and VariantStudio (Illumina), and variant calling was carried out by GATK software. Variants were filtered following standard quality and depth criteria and candidate regions without adequate coverage or quality were amplified by Sanger sequencing. The PredictSNP consensus classifier was used to predict the effect of the amino acid missense substitution identified (15). LOH analysis of the *DLST* locus was performed on tumour DNA from case 1 (see **Figure 1**) by high-density SNP-array analysis. Briefly, a genome-wide scan was conducted on 250 ng of tumour DNA with the Illumina Human610-Quad BeadChip (Illumina) and image data was analysed with the Chromosome Viewer tool contained in GenomeStudio 2010.2 (Illumina). The metric we used was the log-R ratio, which is the binary logarithm of the ratio of the observed to expected normalized R values for a given SNP, as previously reported (16).

### Methylation arrays

Bisulfite conversion of tumour DNA from case 1 (carrying the germline DLST-p.Gly374Glu mutation) was performed using the EZ DNA Methylation Kit (Zymo Research) and genome-wide DNA methylation was assayed using the Infinium MethylationEPIC BeadChip (Illumina) at the Centro Nacional de Genotipado (CEGEN-ISCIII) (www.cegen.org), as previously described (17). This BeadChip interrogates over 850,000 methylation sites per sample at single-nucleotide resolution. M values were used for statistical analyses. Following, we profiled MethylationEPIC data obtained from the tumour with our series of 12 PPGLs carrying mutations in different susceptibility genes (including three cases with the DLST-p.Gly374Glu mutation). Hierarchical clustering of methylation data was performed using GeneCluster 2.0 (18). The methylome of the tumor carrying co-ocurrent mutations in *DLST* and *NF1* (case 1) was deposited in the National Center for Biotechnology Information GEO database under the accession number GSE217207. Methylation data from the rest of the tumor samples used in this study were previously deposited under the accession numbers GSE111336 and GSE123185 (16).

### Liquid chromatographic-tandem mass spectrometric (LC-MS/MS) determination of tricarboxylic acid (TCA) cycle -related metabolites

FFPE tumour tissue (5–10 mg) from three available individuals (cases 1 and 24 with dual *NF1*/*DLST* variants, and case 3 with dual *NF1*/*MDH2* variants) were immersed in 500 mL LC-MS/MS-grade methanol containing isotope-labelled internal standards and processed as previously described (19). An analysis of metabolites was carried out with an AB Sciex 5500 QTRAP mass spectrometer coupled to an Acquity ultra-high-performance liquid chromatographic system (Waters) as previously described (19). We compared the data with that from a series of tumours carrying either *NF1* or *DLST* mutations previously analysed in our laboratory.

### Immunohistochemistry (IHC)

Immunohistochemical staining of DLST (11954; rabbit monoclonal 1:150, Cell Signaling Technology), and 5-hydroxymethylcytosine (5-hmC) (Active Motif; 39770) were performed with 3 mm FFPE sections from tumours 1 and 24, the two *DLST*/*NF1*-mutated tumour samples, as previously described (16, 20).

### RNA sequencing

RNA libraries were built using QuantSeq 3’ mRNA-Seq Library Prep Kit FWD for Illumina (Lexogen, 015), following the vendor’s protocol for FFPE RNA using 500 ng of total RNA from tumours 1 and 24, carriers of dual mutations in *NF1* and *DLST*. We also used UMI Second Strands Synthesis Module for QuantSeq FWD (Lexogen, 081) and PCR Add-on Kit for Illumina (Lexogen, 020) to adjust the library amplification number of cycles. Libraries were applied to an Illumina flow cell for cluster generation and sequenced on NovaSeq6000. For the hierarchical clustering, performed with GeneCluster 2.0 (18), we used expression data from a list of genes differentially expressed in *DLST* mutated PPGLs (16) from the two *NF1*/*DLST* mutated tumours, three *DLST*- and seven *NF1*-mutated PPGLs. The transcriptomes of the tumors carrying co-ocurrent mutations in *DLST* and *NF1* (cases 1 and 24) were deposited in GEO under the accession number GSE217206. RNA-Seq data for the rest of the tumors used in this study were previously deposited under the EGAS00001006044 accession number in the European Genome-phenome archive (Calsina et al., under review).

## Results

### NGS analysis

Amongst the twenty-three germline *NF1* mutations included in the study, three missense variants (identified in cases 3, 15 and 23; **Table 1**; **Figure 1**) were not classified or were classified as variants of uncertain significance by ClinVar, and have not been found in *NF1* patients. However, most of the predictions included in Varsome suggest a pathological effect of the substitutions, so we took these variants into account throughout the current study. Overall, NF1 clinical features (**Table 2**) or a family history of NF1 were reported in ~57% of the individuals carrying *NF1* germline mutations.

Targeted sequencing of a panel of 33 PPGL-related genes revealed three germline variants, one in *DLST* and two in *MDH2*, and two somatic mutations in *H3-3A* (previously known as *H3F3A*) and *PRKAR1A* amongst the twenty-three patients carrying *NF1* germline mutations analysed (**Table 1**; **Figure 1**). The DLST-p.Gly374Glu variant, previously reported as pathogenic in PPGL patients (16, 21), was found in a patient with bilateral PCC. The MDH2*-*p.Lys314Met variant, identified in two unrelated patients, has not been previously described in PPGL patients and it is found in 6/251092 controls from gnomAD. Several software (Polyphen/SIFT, Mutation taster, MutationAssessor, CUPSAT, Mutant v3.0) predicted the variant as deleterious. The *H3-3A* variant, p.Gly35Trp, is a known hotspot mutation found in giant cell tumour of bone (22) and PPGL (23).

Three additional patients carrying *NF1* somatic mutations were also included in the study: one patient with PCC, and family history of NF1, carrying a known *SDHB* germline pathogenic mutation (ClinVar ID:29896; LOVD-SDHB ID: 000047), one patient with a *DLST* pathogenic mutation p.Gly374Glu (ClinVar ID:635133) identified in the germline, and one case with an *ATRX* somatic truncating mutation.

All variants were validated by Sanger sequencing.

### SNP array

Targeted and Sanger sequencing revealed no LOH of the wild-type (WT) *DLST* allele in the two tumours carrying the p.Gly374Glu mutation. For one of the tumours, a genome-wide SNP array analysis confirmed the absence of LOH and discarded uniparental disomy (UPD) of chromosomal region 14q, on which *DLST* is located, a mechanism described in *DLST*-mutated PPGLs (**Supplementary Figure 1**) (16). This tumor showed LOH on the long arm of chromosome 17 including the *NF1* locus.

### Methylation profiling

**Figure 2:**
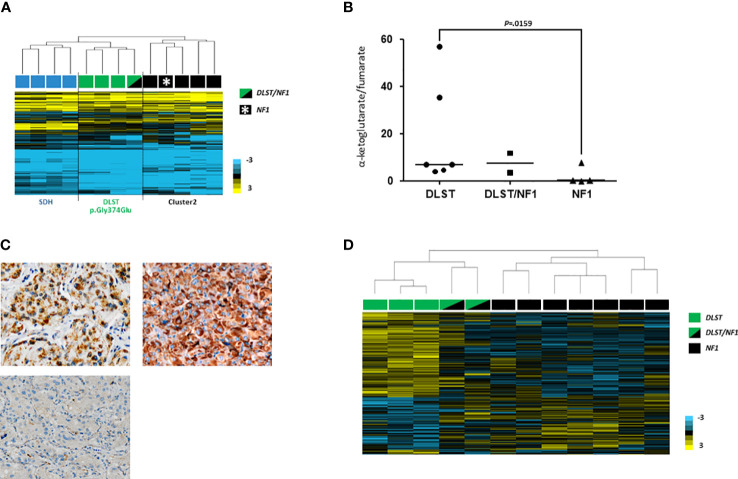
**(A)** Hierarchical clustering performed on the basis of methylation data from PPGLs as follows: one of the two *DLST*/*NF1* (indicated with a green and black rectangle), four SDH-mutated (indicated with blue rectangles), three *DLST*-mutated (indicated with green rectangles) and five cluster 2-mutated (denoted in black rectangles) PPGLs. The DLST/NF1 PPGL was grouped with *DLST*-mutated tumours, and separated from cluster 2-mutated PPGLs. City-block and complete linkage characteristics were used for the analyses. **(B)** α-ketoglutarate/fumarate ratios assessed by LC-MS/MS in *DLST*-mutated tumours (n = 6), *NF1*-mutated PPGLs (n = 4), and the two tumours carrying dual *NF1*/*DLST* mutations. Black lines represent medians. A t test identified significant differences between *DLST*-mutated and *NF1*-mutated tumours. **(C)** Immunostaining for DLST was conducted in the two tumours carrying *DLST*/*NF1* dual mutations (upper panel) revealing the characteristic cytoplasmic aggregates (x20). A VHL-mutated tumor was used as a negative control (lower panel) **(D)** Hierarchical clustering of the two *NF1*/*DLST* mutated tumours, three *DLST*- and seven *NF1*-mutated PPGLs based on gene expression data from a previously reported list of genes differentially expressed in *DLST*-mutated PPGLs. The two tumours carrying the *NF1*/*DLST* mutations were clustered between PPGLs carrying mutations in *NF1* and *DLST*. Uncentered correlation and complete linkage characteristics were used for the analysis.

It has been reported that the methylation profile observed in *DLST*-mutated tumours is intermediate between the CpG island methylator phenotype (CIMP) described for SDH-mutated PPGLs and the unmethylated profile exhibited by tumours belonging to the so called cluster 2 (i.e. tumours with mutations in *NF1*, *RET*, *MAX* or *HRAS*) (24). When we profiled one of the tumours carrying mutations in *NF1* and *DLST*, it clustered together with *DLST*-mutated tumours and separated from cluster 2 samples (including one *NF1*-mutated PCC) (**Figure 2A**), suggesting a role of the *DLST* variant in the observed molecular phenotype.

### Metabolite analysis

LC-MS/MS analysis of the three tumours available did not reveal conclusive alterations in TCA-cycle metabolites. However, one of the tumours carrying the double mutation in *NF1*/*DLST* exhibited an accumulation of cis-aconitate and a slightly high ketoglutarate/fumarate ratio was observed in the two *NF1*/*DLST*-mutated tumours compared to tumours carrying only an *NF1* mutation (**Figure 2B**). It is possible that a certain block in the TCA-cycle due to the *DLST* mutation causes this partial accumulation. Finally, a relatively high fumarate to succinate ratio was observed in the only available tumour carrying dual *NF1*/*MDH2* variants (**Supplementary Figure 2**), something previously described in *MDH2*-mutated PPGL (19).

### IHC assays

**Figure 3:**
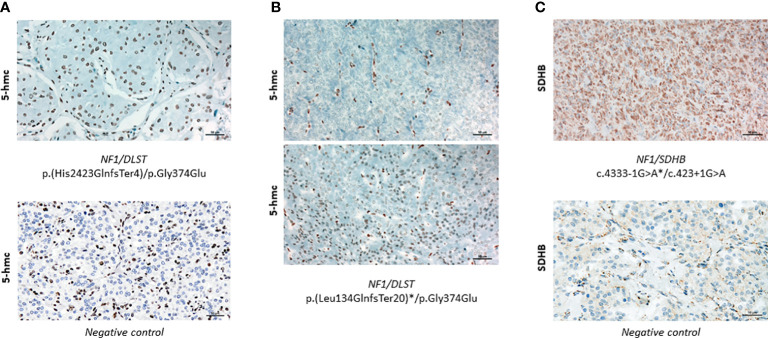
**(A)** Positive 5-hmC IHC performed in case 1 (upper panel) compared to a negative control (lower panel) (x20). **(B)** 5-hmC IHC performed in case 24, showing patches of negative (upper panel) and positive (lower panel) staining (x20)**(C)** Positive SDHB IHC performed in the tumour carrying *SDHB*/*NF1* dual germline mutations (upper panel) compared to a negative control (lower panel) (x20). Asterisks denote a somatic mutation.

In order to investigate whether tumours carrying the *NF1*/DLST-p.Gly374Glu mutations showed other molecular features observed previously in *DLST*-mutated PPGLs, we carried out the IHC for DLST. The two tumours showed a highly positive staining (**Figure 2C**), something previously reported for PPGLs carrying mutations in *DLST* and other TCA-cycle genes (16, 21), supporting again a role of the *DLST* mutation in tumour development. Another molecular marker described for *DLST*-mutated tumours is the low level of 5-hmC nuclear staining (21), similar to the observed in SDH-mutated PPGLs (25). 5-hmC staining applied to the two *DLST*-mutated tumours were positive in one of the samples and heterogeneous in the second tumour, in which positive regions alternated with regions that had negative IHC (**Figuress 3A, B**). SDHB IHC carried out in the tumour carrying co-occurrent *SDHB* germline mutation and somatic *NF1* mutation yielded a positive stain, which is atypical for pathogenic *SDHB* mutations (**Figure 3C**).

### Transcriptional profiling

Hierarchical clustering of the two tumours carrying dual mutations in *NF1* and *DLST* based on expression data for genes differentially expressed in *DLST*-mutated PPGLs grouped them in between *DLST* and *NF1* mutated PPGLs, suggesting that an intermediate phenotype was caused by the coexistence of the two alterations (**Figure 2D**).

## Discussion

PPGL is the tumour with the highest degree of heritability attributable to the presence of germline mutations in known genes (up to 40% of cases) (26). Although mutations in some of the susceptibility genes are highly penetrant (e.g. germline mutations in *VHL* or *RET*), variants in other genes such as *SDHB*, *SDHA* (27) or *TMEM127* (28) do not segregate with the disease in some pedigrees due to a low age-dependent penetrance. In this regard, the latest susceptibility genes identified in PPGL patients*, SLC25A11* and *DLST*, have been found in patients with no relatives affected (16, 29), suggesting that these variants have a very low penetrance as well. Though penetrance of *NF1* germline mutations is considered to be virtually complete after childhood, some studies have reported the presence of unexpected *NF1* germline mutations in PPGL patients for whom a clinical diagnosis of the disease was not previously established (3, 9). The finding of 11 additional PPGL patients harbouring germline *NF1* mutations without clinical manifestations of NF1 at diagnosis, underscores the importance of including *NF1* testing in the genetic diagnosis of every PPGL patient, even in the absence of clinical features of the disease.

The penetrance of a given allele can be modified by the simultaneous presence of variants at other gene loci, which may also affect the clinical expressivity of the disease. In the case of NF1, modifier genes have been reported to contribute to the variable expressivity of the disease in a large series of patients (30, 31). The number of café-au-lait macules has been found to be influenced by common SNPs (32), polymorphisms in *ADCY8* correlate with glioma risk in a sex-specific manner (33), and rare germline variants in various genes including *ATM* have been proposed as candidate modifiers of plexiform neurofibroma (34). NF1-associated PPGLs are mostly unilateral (84% of patients) and rarely extra-adrenal (6.1% of patients) (4, 5). Herein we describe two NF1 patients with multiple tumours harboring dual mutations, which, in addition to the previously reported patients carrying *SDHD*/*NF1* mutations, suggest that the co-occurrence of *NF1* mutations with variants affecting other PPGL susceptibility genes (i.e. *DLST*, *MDH2, SDHD*), or still unknown genes, could be responsible of this unusual clinical manifestation in some NF1 patients. In turn, no germline mutations affecting other PPGL susceptibility gene were found in the six exomes from patients with unilateral PCC and constitutive *NF1* mutation included in The Cancer Genome Atlas program (24). Another possible explanation is that the development of PPGL in NF1 is an example of an oligogenic disease caused by the co-occurrence of mutations in two or more genes. In contrast to the thousands of reports in which mutations in single genes cause human diseases, there are only dozens of human disease phenotypes with evidence of digenic inheritance (35–37). The incidence of NF1, approximately 1:2600 to 3000 individuals, could indicate that the findings described in the present study are coincidental.

The DLST-p.Gly374Glu variant has recently been demonstrated to be pathological in PPGL patients (PGL7; MIM # 618475) (16, 21). Despite the absence of LOH affecting the wild-type allele in the two patients in our series, the highly positive IHC for DLST as a molecular marker of TCA-cycle disruption (16), supports its role in tumour development. In addition, the intermediate expression profile of the two tumours between *NF1* and *DLST* mutated PPGLs, the DLST-like methylation profile observed for one of them, and the accumulation of cis-aconitate and the heterogeneous 5-hmC staining observed in the other tumour, reinforces a role of the *DLST* mutation in tumour molecular characteristics. No additional cases with PPGL were identified in the two pedigrees carrying the DLST-p.Gly374Glu mutation, and the finding of the mutation in a healthy 58-year-old relative from one of the families (data not shown) supports the previously described low penetrance of the DLST-p.Gly374Glu variant (16). Interestingly, the patient carrying the *NF1*/*DLST* variants in the germline (case 1) also developed a medullary thyroid carcinoma (MTC) with LOH for the *NF1* mutation (9). Neither *NF1* nor *DLST* are clearly related to medullary thyroid malignancies, but the co-occurrence of both alterations could account for the presence of bilateral PCC and MTC in the same patient, resembling what happens in multiple endocrine neoplasia type 2. This case is especially intriguing, as clinical features of NF1 were not recognized until the diagnosis of bilateral PCC was made at age 56 years, and in addition the patient developed primary prostate and lung cancers during his lifetime.

The *MDH2* variant, p.Lys314Met, identified in two of the NF1 patients involves one of the four lysines described as playing a role in the activation of MDH2 by acetylation (38, 39). Interestingly, the same lysine was found deleted in another patient with multiple noradrenergic PGLs (40), which supports the relevance of this residue for MDH2 function. The presence of a para-aortic PGL in one of the patients carrying the p.Lys314Met (case 3), reinforces the role of the variant in the disease since a thoracic-abdominal location of PGL, very rare in NF1 patients, is the most frequently found in *MDH2* mutation carriers (19, 40).

The presence of *NF1* somatic mutations is one of the main genetic findings occurring in sporadic PPGLs (accounts for 20-25% of all cases) (41), being mostly tumours with adrenal location and only 4% extra-adrenal (10, 42). It has been repeatedly suggested that there is a mutual exclusivity condition affecting driver events in PPGLs, something that points to the redundancy of the affected signals (43, 44). Finding a mutation in *H3-3A* in a tumour carrying a germline *NF1* variant suggests that perhaps some mutations in *NF1* do need another alteration that confers additional selective advantages to the tumour cells (44). In the majority of NF1 cases, the mutation in *NF1* is sufficient for tumour development and there is no need for accumulation of mutations in other predisposing genes, although it has been suggested that a proportion of apparently NF1-sporadic PPGLs might actually have a germline susceptibility variant in other predisposing genes (44). This is the case of the patient described in the present study carrying a germline *SDHB* mutation and a somatic *NF1* alteration, whose tumour showed no evidence of LOH and a positive SDHB IHC. There is another similar example of co-occurrence of a germline *SDHB* mutation and a somatic *NF1* alteration in a patient who developed a para-aortic PGL (45). Recently, dual *SDHB*/*NF1* loss has been reported as a successful mechanism to force tumour formation in a *SDHB* knockout mouse model (46). *SDHB* loss has been shown to be insufficient for tumour development in SDH-mouse models of PPGL (47), but the concomitant alteration of NF1 finally yielded SDH-like PPGLs, reinforcing the theory for a need of additional genetic events for tumour initiation and maintenance in *SDHB* PPGL, resulting in a lower penetrance of the mutations (48). A similar situation could explain the low penetrance of the p.Gly374Glu *DLST* germline variant observed in our pedigrees. It is noteworthy that all the NF1 cases described herein, as well as those reported in the literature, carry co-occurrent alterations in an apparently unrelated pathway (e.g. pseudohypoxic signalling), suggesting that co-occurrence of alterations in different signalling cascades could provide a selective advantage for the development of PPGL.

Somatic *ATRX* mutations in PPGLs carrying germline mutations in one of the major susceptibility genes (especially in *SDHB*-mutated cases) have been reported (24, 49), and this event has been described as an independent factor of poor prognosis (50). Moreover, a recent study described the co-existence of *NF1* germline mutations and *ATRX* somatic alterations in different tumours from NF1 patients (51). Therefore, finding a PPGL carrying somatic mutations in *NF1* and *ATRX* may not be unexpected, and warns of the malignant potential of the tumour carrying the *ATRX* mutation. Finally, *PRKAR1A*, mapped at 17q22-24, is the gene encoding the protein kinase A regulatory subunit type 1α which can interfere with the ERK1/2 cascade of the MAPK pathway and causes inhibition of cell proliferation (52). *PRKAR1A* is frequently mutated in patients with Carney complex syndrome (MIM #160980) (53), a rare multiple neoplasia syndrome characterized by both endocrine and non-endocrine manifestations. This gene can be rearranged with *RET* to form the thyroid tumour-specific chimeric oncogene PTC2 (54), and it is worth noting that dysregulated protein kinase A has been linked to cancer by activation of mechanisms that overlap but differ from those found in neurofibromatosis tumorigenesis (55). *PRKAR1A* haploinsufficiency is a general tumorigenic signal which may require inactivation of other tumour suppressors to function (56). Another inactivating variant in *PRKAR1A* has been found in a metastatic PCC carrying additional alterations in *ATRX* and *MAML3* (24), but the significance of this somatic event in PPGL etiology is still unknown.

Because of the low penetrance of some mutations, variable expressivity, and parent-of-origin effects associated with some susceptibility genes, a large number of PPGL patients have no family history, masking the hereditary condition and making proper follow-up of individuals at risk for hereditary PPGL difficult. Herein, we propose a new layer of complexity in which mutations affecting genes involved in different pathways (e.g. pseudohypoxic and receptor tyrosine kinase signalling) (**Supplementary Figure 3**) co-occurring in the same patient, either in the germline or in the tumour, could provide a selective advantage for the development of PPGL. The widespread application of NGS to the diagnosis of PPGL will continue to identify unexpected co-occurrences of pathological mutations, which may provide new insights into the development of these tumors, as well as explain differences in their expressivity and incomplete penetrance.

## Data availability statement

The data presented in the study are deposited in the GEO database repository, accession numbers GSE217208, GSE111336 and GSE123185; and in the in the European Genome-phenome archive, accession number EGAS00001006044.

## Ethics statement

The studies involving human participants were reviewed and approved by Institutional Review Board of Mayo Clinic: IRB 13-004137; Cambridge East Medical Research Ethics Committee: Ref: 06/Q0104/133; Hospital Universitario 12 de Octubre: 15/024; ENS@T Ethics Committee: 88/11; Azienda Ospedaliera Universitaria Careggi: Prot. N. 2011/0020149; Ethics Committee Institutului National de Endocrinologie: 14/11.06.2020; The Instituto de Salud Carlos III (ISCIII) ethics committee: CEI PI 54_2016-v2. The patients/participants provided their written informed consent to participate in this study.

## Author contributions

SM, EG, RL, EC, SR, GE, and EH were involved in data analysis, data interpretation, and final approval of the manuscript. NP, ML, JG, AL-F, MC, AH-M, MG, XM-G, MB, EK, EL, FM, SL, SF, NB, LC, ER, and IB were responsible for data collection and final approval of the manuscript. MR was responsible for study design, data interpretation, manuscript writing, and final approval of the manuscript. AC was responsible for study design, data analysis, data interpretation, manuscript writing, and final approval of the manuscript. All authors contributed to the article and approved the submitted version.

## Supplementary material

Supplementary Figure 1 SNP-array study of chromosomes 14 and 17 using tumour DNA from case 1 who carries the DLST-p.Gly374Glu/NF1-p.His2423GlnfsTer4 dual mutation showed no LOH. The lower panel shows the genomic plots of the log R ratio [log 2 (Rpatient/Rreference)], and the upper panel gives the allele frequency parameters along the chromosomes.

Supplementary Figure 2 Fumarate/succinate ratios assessed by LC-MS/MS in *MDH2*-mutated PPGLs (n=5), *NF1*-mutated PPGLs (n=5), SDHx-mutated PPGLs (n=11), and one PPGL carrying dual *NF1*/*MDH2* mutation. Black lines represent means. A t test identified differences between means.

Supplementary Figure 3 Schematic representation of the three molecular processes involved in the development of PPGL in which we have found co-occurring mutations: pseudohypoxic signalling (shown in purple), receptor tyrosine kinase cascades (shown in green), and epigenetic modifications and chromatin remodelling (shown in blue). The proteins found mutated in PPGL are indicated with dark coloured ovals and white letters, and the proteins whose genes have been found mutated in the present study are indicated with a yellow asterisk.
