# Supplementary figures and images for: Co-occurrence of mutations in NF1 and other susceptibility genes in pheochromocytoma and paraganglioma
Source: Front Endocrinol (Lausanne). 2023 Jan 25;13:1070074. doi: 10.3389/fendo.2022.1070074 (PMC9905101; doi:10.3389/fendo.2022.1070074)

## Slide 1
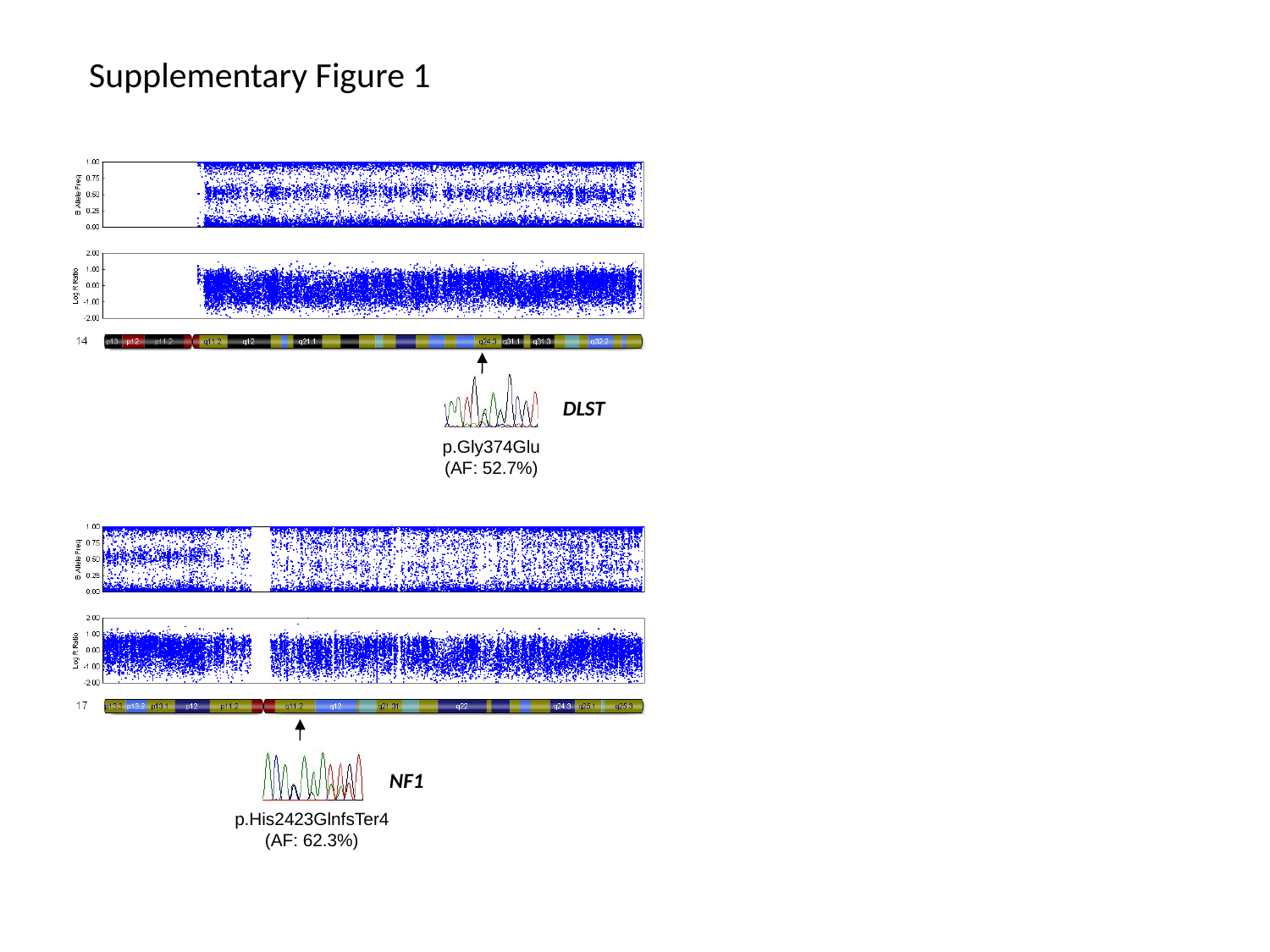

Supplementary Figure 1
DLST
p.Gly374Glu (AF: 52.7%)
NF1
p.His2423GlnfsTer4 (AF: 62.3%)

Supplement: Supplementary Figure 1 — SNP-array study of chromosomes 14 and 17 using tumour DNA from case 1 who carries the DLST-p.Gly374Glu/NF1-p.His2423GlnfsTer4 dual mutation showed no LOH. The lower panel shows the genomic plots of the log R ratio [log 2 (Rpatient/Rreference)], and the upper panel gives the allele frequency parameters along the chromosomes. [file Presentation_1.ppt]

## Slide 1
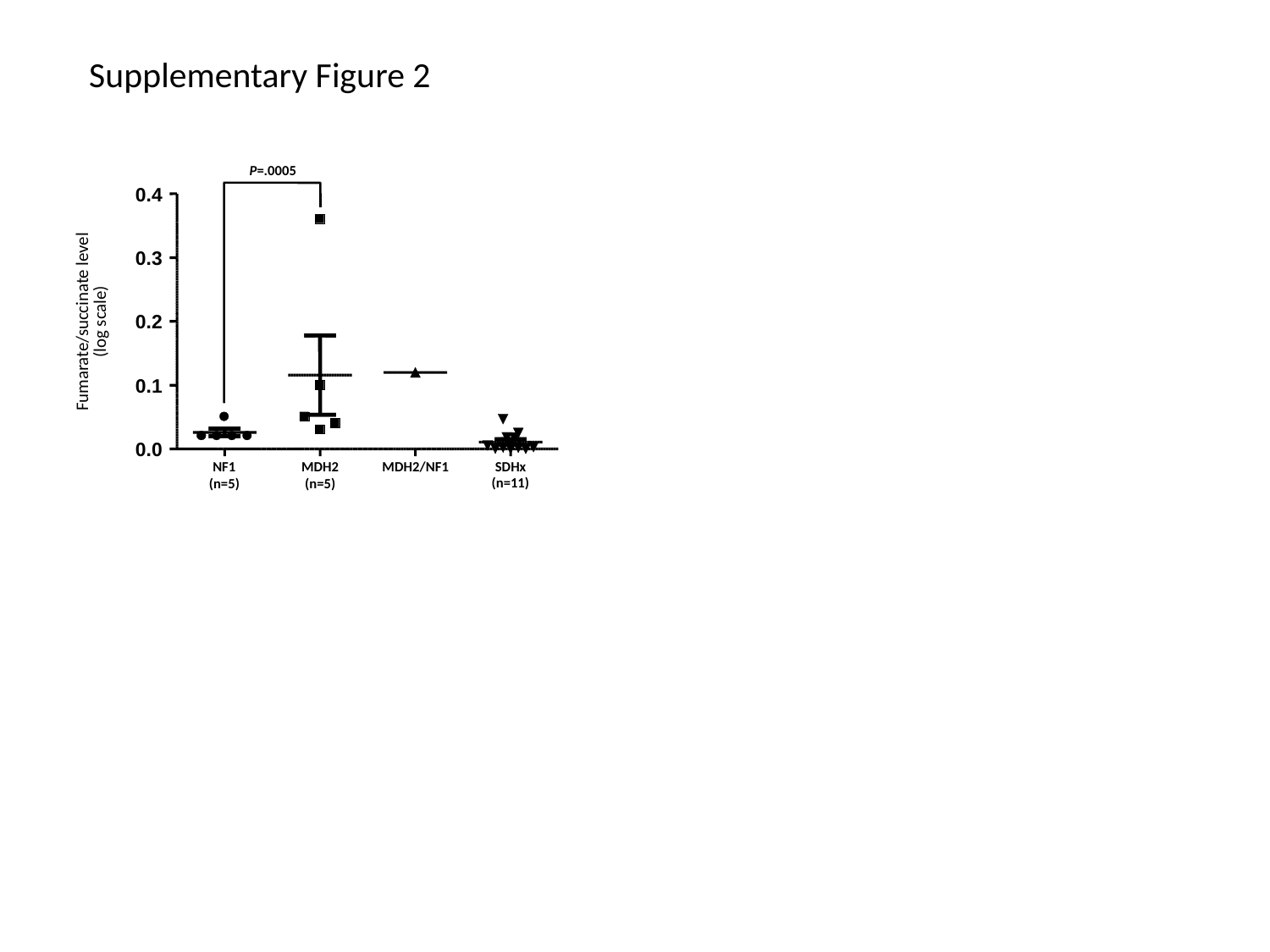

Supplementary Figure 2
P=.0005
0.4
0.3
0.2
0.1
0.0
Fumarate/succinate level (log scale)
SDHx
(n=11)
NF1
(n=5)
MDH2
(n=5)
MDH2/NF1

Supplement: Supplementary Figure 2 — Fumarate/succinate ratios assessed by LC-MS/MS in MDH2-mutated PPGLs (n=5), NF1-mutated PPGLs (n=5), SDHx-mutated PPGLs (n=11), and one PPGL carrying dual NF1/MDH2 mutation. Black lines represent means. A t test identified differences between means. [file Presentation_2.ppt]
