# Supplementary material for: Co-occurrence of mutations in NF1 and other susceptibility genes in pheochromocytoma and paraganglioma
Source: Front Endocrinol (Lausanne). 2023 Jan 25;13:1070074. doi: 10.3389/fendo.2022.1070074 (PMC9905101; doi:10.3389/fendo.2022.1070074)
Supplement: Supplementary Figure 3 — Schematic representation of the three molecular processes involved in the development of PPGL in which we have found co-occurring mutations: pseudohypoxic signalling (shown in purple), receptor tyrosine kinase cascades (shown in green), and epigenetic modifications and chromatin remodelling (shown in blue). The proteins found mutated in PPGL are indicated with dark coloured ovals and white letters, and the proteins whose genes have been found mutated in the present study are indicated with a yellow asterisk. [file Presentation_3.pptx]

## Slide 1
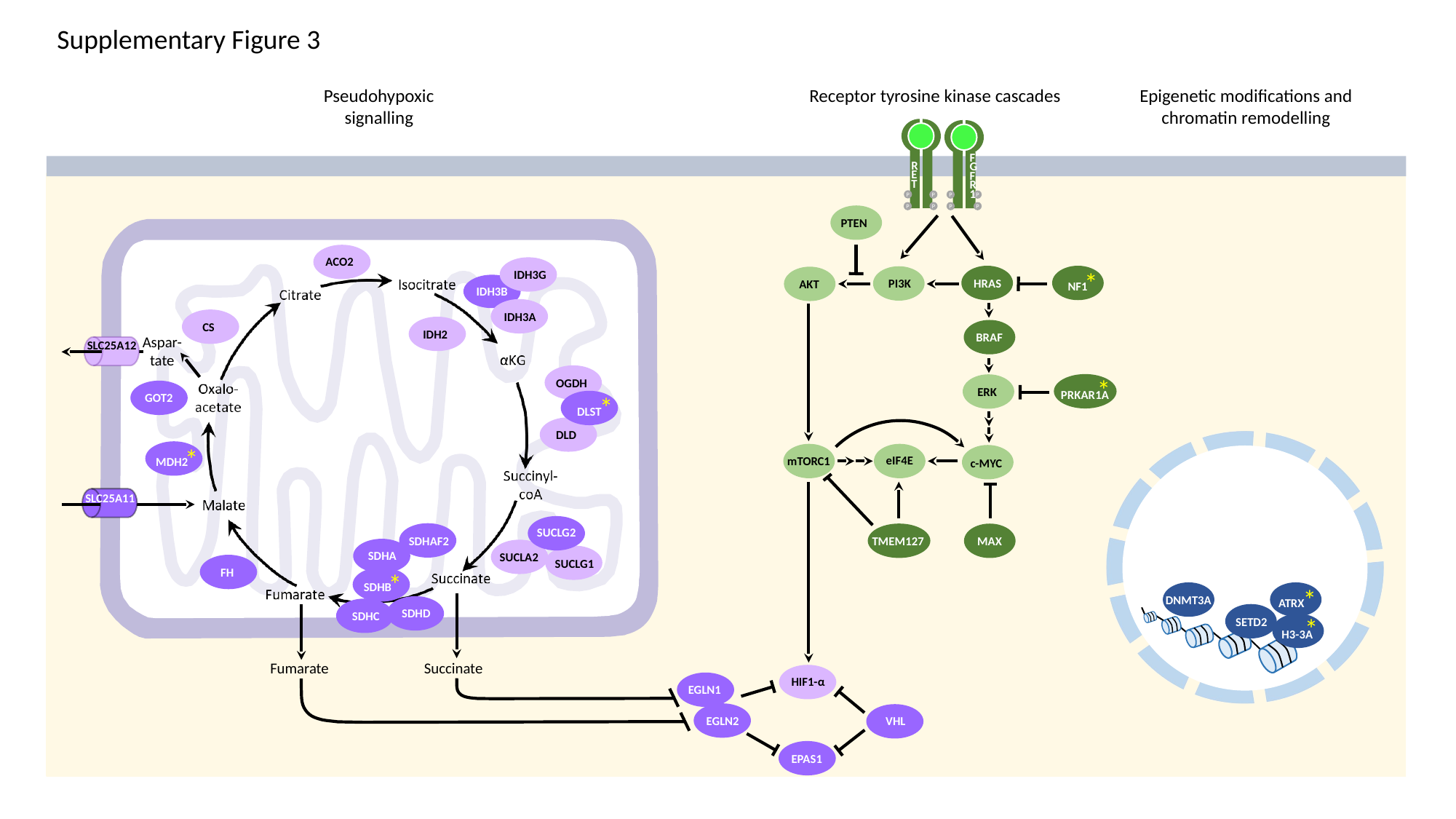

Supplementary Figure 3
Receptor tyrosine kinase cascades
Pseudohypoxic signalling
Epigenetic modifications and chromatin remodelling
R
E
T
P
P
P
P
F
G
F
R
1
P
P
P
P
PTEN
ACO2
IDH3B
CS
IDH2
OGDH
DLST
DLD
MDH2
SUCLG2
SDHAF2
SDHA
SUCLA2
SUCLG1
FH
SDHB
SDHD
SDHC
*
IDH3G
HRAS
NF1
PI3K
AKT
IDH3A
BRAF
Aspar-
tate
SLC25A12
*
PRKAR1A
ERK
*
GOT2
*
mTORC1
eIF4E
c-MYC
SLC25A11
TMEM127
MAX
*
*
DNMT3A
ATRX
SETD2
*
H3-3A
Succinate
Fumarate
HIF1-α
EGLN1
EGLN2
VHL
EPAS1
